# Multienergy cardiovascular CT imaging: current state and future

**DOI:** 10.1093/bjr/tqae246

**Published:** 2024-12-05

**Authors:** Konstantin Klambauer, Costanza Lisi, Lukas Jakob Moser, Victor Mergen, Thomas Flohr, Matthias Eberhard, Hatem Alkadhi

**Affiliations:** Diagnostic and Interventional Radiology, University Hospital Zurich, University of Zurich, 8091 Zurich, Switzerland; Diagnostic and Interventional Radiology, University Hospital Zurich, University of Zurich, 8091 Zurich, Switzerland; Department of Biomedical Sciences, Humanitas University, 20090 Milan, Italy; Diagnostic and Interventional Radiology, University Hospital Zurich, University of Zurich, 8091 Zurich, Switzerland; Diagnostic and Interventional Radiology, University Hospital Zurich, University of Zurich, 8091 Zurich, Switzerland; Diagnostic and Interventional Radiology, University Hospital Zurich, University of Zurich, 8091 Zurich, Switzerland; Department of Radiology and Nuclear Medicine, Maastricht University Medical Centre, 6229 Maastricht, The Netherlands; Diagnostic and Interventional Radiology, University Hospital Zurich, University of Zurich, 8091 Zurich, Switzerland; Diagnostic and Interventional Radiology, University Hospital Zurich, University of Zurich, 8091 Zurich, Switzerland

**Keywords:** computed tomography, photon-counting detector, multienergy imaging, cardiovascular, contrast media

## Abstract

Multienergy cardiovascular CT imaging can be defined as data acquisition at 2 (dual-energy) or multiple X-ray energies. Multienergy cardiovascular CT imaging provides additional qualitative and quantitative information such as material maps or virtual monoenergetic images, which are supposed to further improve the quality and diagnostic yield of CT. Recently introduced photon-counting detector CT scanners further address some of the challenges and limitations of previous, conventional CT machines, hereby enhancing and extending the applications of CT for cardiovascular imaging. This review summarizes the technical principles of multienergy cardiovascular CT imaging and addresses the optimization of image quality and discusses the various dual-energy-based applications for coronary, valvular, and myocardial imaging. New developments in regard to k-edge imaging and new contrast media for multienergy cardiovascular CT imaging are being also discussed.

## Introduction

Multienergy CT imaging is defined as CT data acquisition at 2 (dual-energy) or multiple X-ray energies. Multienergy cardiovascular CT imaging provides additional quantitative information such as material maps or virtual monoenergetic images (VMIs), which are supposed to further improve the quality and diagnostic yield of the examination. Photon-counting CT (PCD-CT), the most recent technique, is a major step forward in multienergy cardiovascular CT imaging.^1^^,^^2^ This review article is aimed to provide an overview of major technical aspects and various advantages and application options of multienergy cardiovascular CT imaging with a focus on the optimization of the image quality through low- or high-energy VMIs, the dual-energy-based options of virtual non-contrast/non-iodine and virtual non-calcium cardiovascular imaging, and the added value of iodine mapping of the myocardium in late enhancement CT. In addition, the article tries to shed light on future developments in regard to k-edge imaging and new contrast media for multienergy cardiovascular CT imaging.

## Multienergy CT: technical aspects

Established CT systems are limited to data acquisition at 2 different energies (dual-energy CT), regardless of the respective technical realization.^3^ Dual-energy CT enables differentiation of one material with a relatively low atomic number Z (such as water or soft tissue) from another one with a relatively high Z (such as calcium or iodine).^3^ This is the basis for the computation of virtual non-contrast (VNC) images and iodine maps, non-iodine (VNI) and non-calcium images (VNCa), and VMIs.

Dual-source CT systems operate both X-ray tubes at different tube voltages (eg, 80 and 140 kV) to acquire dual-energy data.^4^ Optimization of the spectral separation is possible by additional pre-filtration of the high-kV beam.^5^ However, the data of both measurement systems are not perfectly aligned, cross-scattered radiation is a challenge, and the temporal resolution in dual-energy operation is reduced to that of a corresponding single source CT.^4^

In CT systems with fast kV-switching, the X-ray tube voltage is rapidly switched between 80 and 140 kV, such that consecutive projections of a CT scan are acquired at different kV. Low- and high-kV data are well aligned, but temporal resolution suffers from longer gantry rotation times,^3^ spectral optimization is not possible, and anatomical tube current modulation is currently not available.^3^^,^^6^

In CT systems with dual-layer detectors, the upper layer mainly absorbs the low-energy X-rays of the 120 or 140 kV spectrum. In contrast, the lower layer registers the remaining higher-energy X-rays. Dual-energy data acquisition is routinely integrated without special protocols, and data are perfectly aligned.^6^ The spectral separation, however, is inferior to approaches using different X-ray tube voltages.^3^^,^^6^ Another approach to dual-energy imaging is the use of the split-filter technology, which divides the X-ray beam into 2 distinct energy ranges using separate filters placed on either side of the X-ray source. This method enables dual-energy imaging with a single X-ray tube and detector system, though it may result in reduced overall X-ray dose reaching the detector.^7^ However, because of inferior temporal resolution the split-filter option cannot be used for cardiac imaging.

Due to these shortcomings, multienergy cardiovascular CT imaging has so far been mainly limited to technical evaluation and feasibility studies. This may change with the new CT systems with PCD, which have the potential to overcome previous limitations.^3^^,^^8^ PCDs convert the absorbed X-ray quanta directly into current pulses with a height proportional to the X-ray energy. PCDs provide data with high spatial resolution at improved radiation dose efficiency, with better contrasts and spectral separation into 2 or more energy ranges. With dual-source PCD-CT, multienergy imaging became routine in daily clinical work, which is particularly beneficial in cardiovascular imaging because of the retained good temporal resolution (currently 66 ms).^8^ An overview of the various dual-energy CT options is provided in Table 1.

**Table 1.** Technical overview of multienergy CT options.

Options	Description	Comments
Fast kV-switching	Alternates rapidly between 2 kV settings (eg, 80 kV and 140 kV) within a single scan^3^^,^^6^	Provides well-aligned dual-energy data with reasonable spectral separation, but limited temporal resolution and no tube current modulation^3^
Split filter CT	A split filter is used to divide the X-ray beam into 2 energy ranges by placing different filters on either side of the X-ray source^7^	Provides dual-energy imaging with a single X-ray tube and detector setup, offering improved spectral separation; however, the split filter approach may reduce the overall X-ray dose reaching the detector^7^. No application in cardiac imaging.
Dual-layer CT	Two detector layers simultaneously capture low and high-energy X-rays from a single X-ray tube (eg, 120 kV)^3^^,^^6^	Delivers well-aligned dual-energy data without the need for special protocols but offers lower spectral separation compared to other technologies^3^^,^^6^
Dual-source CT	Uses 2 X-ray tubes with different kV settings (eg, 80 kV and 140 kV) for simultaneous imaging^4^^,^^5^	Offers the best spectral separation; cross-scatter radiation must be handled^4^^,^^5^
Photon-counting detector CT	Converts X-ray photons directly into electrical signals with energy resolution for each photon^2^^,^^8^	Inherent spectral image acquisition at the highest available temporal resolution^2^^,^^8^

## Virtual monoenergetic imaging

### Low monoenergetic images to improve contrast-to-noise ratio and reduce contrast media

**Figure 1.**
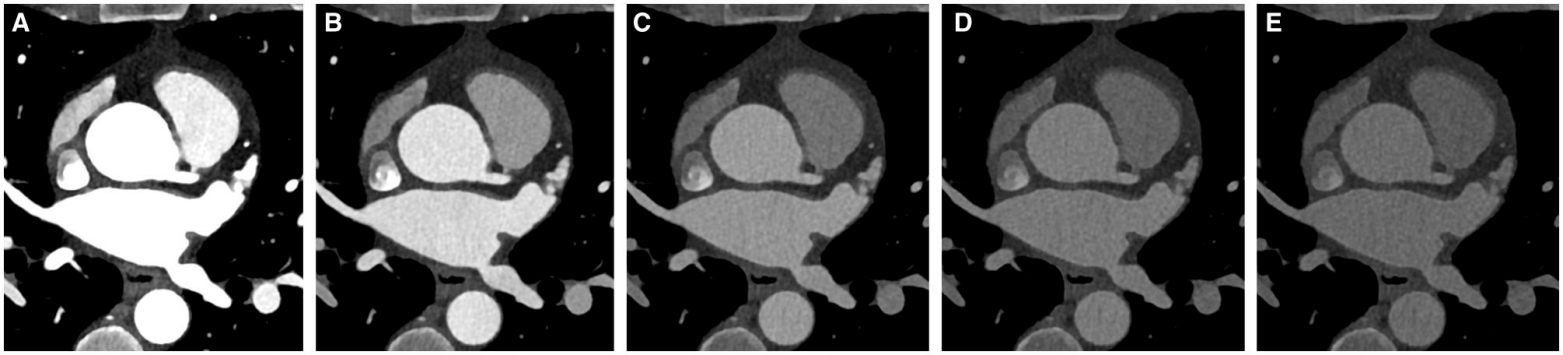
A 47-year-old female patient undergoing multienergy coronary CT angiography with a dual-source photon-counting detector CT (window width: 320; window level: 230). Axial virtual monoenergetic image reconstructions at the origin of the left main artery showing progressively decreasing contrast-to-noise (CNR) ratios at increasing monoenergetic levels, namely 40 keV (**A**), 60 keV (**B**), 80 keV (**C**), 100 keV (**D**), and 120 keV (**E**).

In recent decades, we witnessed continuous reductions of the administered dose of contrast media due to technical developments such as, for example, low tube voltage CT scanning. Further improvements of the contrast-to-noise ratio (CNR) would further enhance the possibility to minimize contrast media administration to our patients. One of the main beneficial aspects of multienergy cardiovascular CT imaging is the increase in the iodine CNR, a crucial factor in vascular pathology detection and characterization.^9^^,^^10^ VMI reconstructions open the possibility of tuning imaging to the clinical requirements of improved contrast resolution and decreased artefacts and noise, which may require different keV levels.^11^^,^^12^ Iodine CNR increases when VMI energies approach the k-edge of iodine.^9^ Consequently, low-energy VMI (ie, <70 keV) is associated with enhanced contrast resolution in cardiovascular CT,^13^^,^^14^ which is even more beneficial when scanning obese patients.^15^ A CNR increase of about 40% was reported by Sartoretti et al^16^ for 40 keV VMI compared to 70 keV VMI, independent of the level of iterative reconstruction. The potential noise increase in low-energy VMIs, a limiting factor in early studies, is meanwhile mitigated by advanced image processing (Mono+).^17^^,^^18^ For these reasons, VMI reconstructions nowadays represent the standard for image reading with PCD-CT.^19^ The impact of reconstruction energy level of VMIs on CNR is exemplified in Figure 1.

Enhanced iodine contrast in low-energy VMIs may be exploited to reduce the total iodine load administered without decreasing diagnostic accuracy, especially in patients where renal function is a concern.^20^ Several contrast media reduction protocols have already been suggested for conventional energy-integrated detector CT operating in dual-energy mode using low-energy VMI reconstructions.^21^^,^^22^ The improved CNR of PCD-CT particularly in vascular imaging^13^^,^^14^^,^^16^ opened to the possibility to lower contrast media volume administration. Non-inferior image quality compared to EID-CT was demonstrated by Higashigaito et al^23^ in CT angiography of the aorta with PCD-CT at 25% lower contrast media volume administration. Similar findings were found *ex vivo* for coronary CT angiography in a dynamic vessel phantom, where VMI reconstructions at 40 keV on dual-source PCD-CT allowed for reducing the contrast media concentration up to 50% with acceptable vessel attenuation in VMI.^24^  *In vivo* studies further strengthened this evidence, showing that PCD-CT enables a considerably reduced contrast media volume administration in coronary CT angiography by 40% while maintaining a diagnostic image quality.^25^ Rajiah et al^26^ evaluated the benefit of low kilovoltage VMI reconstructions for high-pitch coronary CT angiography while applying a low volume of contrast media (30 ml) and found diagnostic quality coronary CT angiography images at both low radiation and low iodinated contrast doses.

### High monoenergetic images to reduce calcium blooming

Coronary CT angiography is the primary non-invasive technique for ruling-out coronary artery disease due to its excellent sensitivity and negative predictive value.^27–29^ As a limitation, however, it is characterized by a lower specificity and positive predictive value^30^ which is mainly attributed to calcified plaques and associated blooming artefacts.^31^^,^^32^ One proposed remedy for this issue is the implementation of VMIs at high energy into the diagnostic workflow^33^ which is most promising for dual-source PCD-CT, because spectral information in coronary CT angiography is acquired at maintained high temporal resolution.^34^

**Figure 2.**
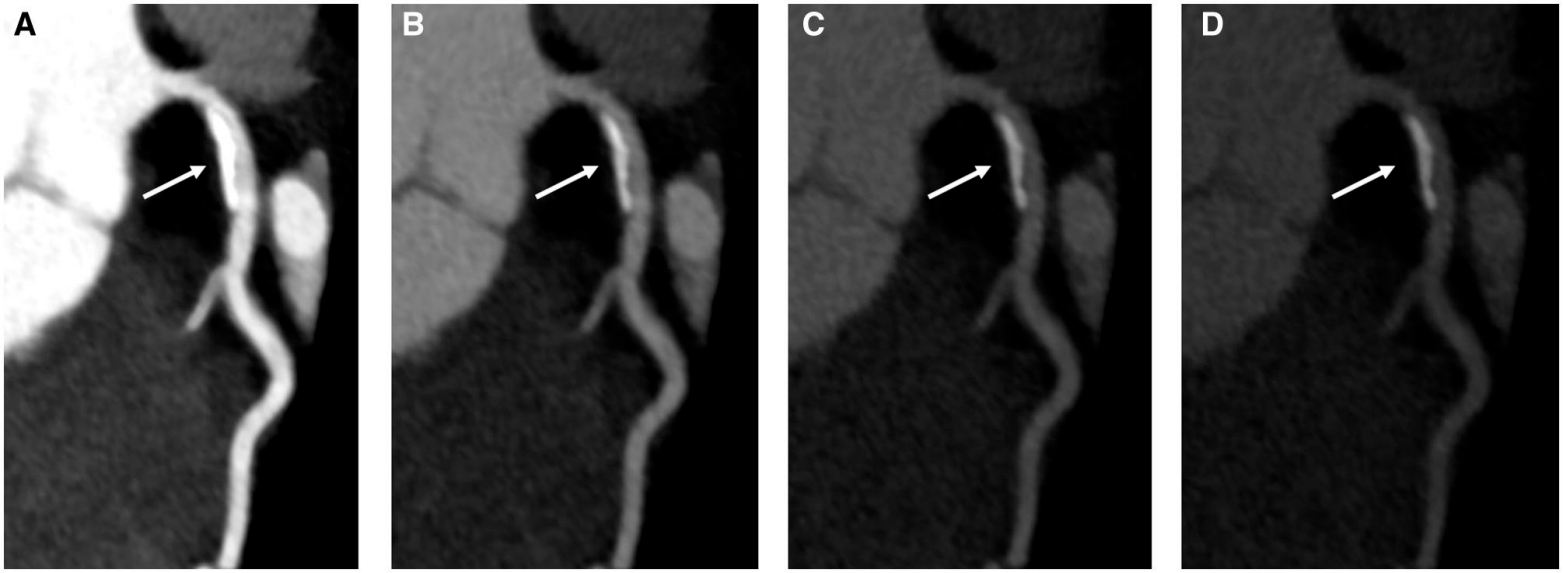
A 56-year-old female patient undergoing multienergy coronary CT angiography with a dual-source photon-counting detector CT. Calcified plaque in the proximal left anterior descending (LAD) artery (arrows) in 40 keV (**A**), 60 keV (**B**), 80 keV (**C**), and 100 keV (**D**) keV reconstructions. Note the reduction of calcium blooming at higher monoenergetic levels and the reduced iodine contrast.

Mid to high energy VMI show reduced blooming artefacts surrounding highly attenuating structures such as calcifications or metal.^33^^,^^35^ Wolf et al^36^ investigated the impact of PCD-CT-derived VMI at different energy levels on coronary calcium blooming and coronary stenosis quantification. Authors reconstructed VMI at 9 energy levels between 40 and 140 keV for 33 patients with 64 coronary stenoses. Compared to the reference standard invasive catheter coronary angiography the authors found best agreement of percentage diameter stenosis for calcified plaques at 100 keV, for mixed plaque at 140 keV, and for non-calcified plaques at 40 keV. The average bias of percentage diameter stenosis for calcified plaques at 100 keV was 17%, and blooming decreased from 40 to 140 keV VMI. Figure 2 illustrates how calcium blooming artefacts are reduced at higher energy VMIs.

Stehli et al^37^ studied a rapid kV switching dual-energy system, analysing 8 VMI energy levels from 50 to 140 keV. Comparing with invasive catheter coronary angiography, they found the best stenosis quantification for calcified and mixed plaques at 90 keV and for non-calcified plaques at 140 keV. The study also noted that calcified and mixed plaques were generally overestimated, while non-calcified plaques were underestimated.

Xu et al^38^ evaluated VMI energy levels between 70 and 140 keV for the quantification of calcified and mixed plaques relative to invasive catheter coronary angiography. They showed a VMI energy level of 100 keV for stenosis quantification was in best agreement with invasive catheter coronary angiography.

In contrast to these relatively high VMI energy levels reported as optimal for calcified plaques, Sartoretti et al^39^ recently found the lowest mean error of stenosis quantification of calcified plaques at a VMI energy level of 65 keV comparing to 3D quantitative invasive catheter coronary angiography. In that study conducted on a dual-source PCD-CT, blooming artefacts decreased from energy levels of 45 to 145 keV.^39^ Obviously, further work on that topic is required before there is consensus on the optimal reconstruction for different clinical scenarios.

## Virtual non-iodine/virtual non-contrast and virtual non-calcium images

Spectral cardiac imaging enables to perform material decomposition of, for example, iodine and soft tissue, or iodine and calcium. VNC or VNI images with removal of iodine and VNCa images with removal of the calcium can be reconstructed. The VNC and VNI option holds promise to obviate the need of true non-enhanced acquisitions for reducing the radiation dose of the examination.^40^ The VNCa option can be important to reduce the effect of calcium blooming artefacts on vessel evaluation, hereby reducing an overestimation of stenosis degrees.^41^ Depending on the specific imaging task, 2 distinct virtual iodine subtraction algorithms are available. The conventional VNC algorithm is based on a material decomposition into iodine and soft tissue. It preserves accurate attenuation values in parenchymatous organs,^40^ but calcium attenuation is reduced. The VNI algorithm is tailored to better preserve the attenuation of calcifications and is therefore recommended for calcium scoring.^40^^,^^42^^,^^43^ This distinction is crucial as iodine and calcium show similar energy dependence of the attenuation, which complicates accurate material separation. In a study by Emrich et al,^44^ coronary calcium scores determined on VNI of coronary CT angiography demonstrated a high correlation, but significant underestimation compared with scores determined on true non-contrast scans. Mergen et al^40^ investigated the accuracy of cardiovascular calcium scores using calcium-preserving VNI from late enhancement scans and found similar calcium scores for the aortic valve, mitral annulus, and coronary arteries compared with true non-contrast scans. Notably, the optimal virtual monoenergetic levels of the calcium-preserving VNI images varied depending on the cardiovascular structures assessed.^40^

**Figure 3.**
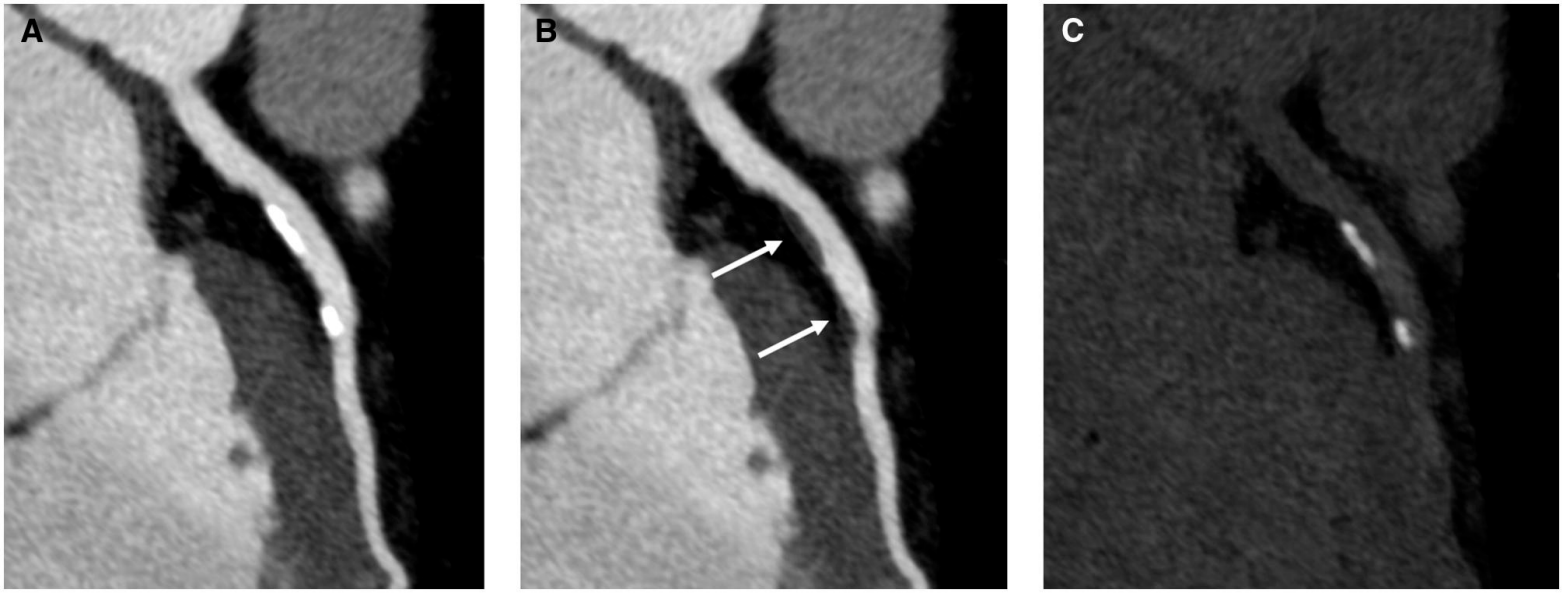
A 53-year-old male patient with increased cardiovascular risk profile and recurrent episodes of atypical chest pain undergoing dual-source photon-counting detector CT. Curved planar reformations of conventional (**A**), virtual non-calcium (**B**), and virtual non-contrast (**C**) images show 2 calcified plaques in the proximal left anterior descending (LAD) artery (arrows). Note the dual-energy-based removal of calcium (arrows) in the virtual non-calcium and the dual-energy-based removal of iodine in the virtual non-contrast image.

In addition to the functionality of the VNC/VNI algorithm, the spectral information included in the cardiovascular PCD-CT scans allows for the dedicated subtraction of calcified plaques. An early phantom study by Allmendinger et al^45^ demonstrated that using a calcium-removal algorithm effectively reduced blooming artefacts caused by heavily calcified plaques and improved image interpretability. Subsequent initial patient experience indicated more accurate quantification of calcified stenoses using virtual non-calcium images of coronary CT angiographies, while stenoses were overestimated using routine VMI compared with quantitative invasive catheter coronary angiography.^39^ Similarly, Nishihara et al^46^ have shown that VNCa images have improved subjective image quality and diagnostic performance of coronary stenoses. Importantly, VNCa images should only be used in conjunction with conventional VMIs, as erroneous plaque subtraction on VNCa images may occur, potentially leading to false diagnoses.^41^ However, when feasible, the virtual non-calcium algorithm has the potential to refine the quantification of calcified stenoses. Examples for VNC, VNI, and VNCa images are shown in Figure 3.

Notably, 2 scan modes are available when acquiring coronary CTAs with PCD-CT: the ECG-gated spectral mode and the ECG-gated ultra-high-resolution mode. Ultra-high-resolution coronary CT angiographies excel at visualizing coronary arteries with a high calcium burden^47^^,^^48^ and assessing patency of coronary artery stents.^43^^,^^48^^,^^49^ However, acquiring cardiovascular scans with both spectral and ultra-high resolution has only recently been introduced, and clinical experience of this combination in ECG-gated cardiovascular CT is still missing.

## Iodine mapping for late enhancement and extracellular volume quantification

With conventional dual-source CT scanners, DE acquisitions of the heart could be performed only when sacrificing the high temporal resolution of the dual-source system. PCD-CT enable spectral data acquisition at the highest temporal resolution of the scanner. In addition, the spectral acquisition of the late iodine enhancement phase obviates the need of a separate non-enhanced scan, which positively impacts also the robustness of the extracellular volume (ECV) mapping technique.^50^^,^^51^

Expansion of the extracellular matrix is a common result of myocardial pathologies like fibrotic collagen accumulation, amyloid deposition, and oedema.^52–54^ Traditionally assessed via invasive endomyocardial biopsy, non-invasive MRI-based ECV quantification is increasingly being used in diagnosing various cardiovascular disease.^55^^,^^56^ Recently, CT-based ECV quantification (ECV_CT_) has emerged as an alternative due to its broader availability, lower costs, and faster image acquisition, despite challenges like reduced soft tissue contrast and ionizing radiation exposure.^57^^,^^58^

ECV_CT_ has demonstrated prognostic value across a spectrum of cardiovascular conditions. Elevated ECV_CT_ is linked to increased risks of heart failure hospitalization, mortality, and reduced left ventricular mass in aortic stenosis patients.^59^^,^^60^ Vignale et al and others have correlated ECV_CT_ with stroke risk, left ventricular ejection fraction, and New York Heart Association (NYHA) class post-valve replacement.^61–63^ ECV_CT_ predicts major cardiovascular events in dilated cardiomyopathy and aids in diagnosing acute myocarditis, as demonstrated by Baggiano et al.^51^^,^^64^ ECV_CT_ also shows promise in identifying myocardial injury in acute coronary syndrome patients with elevated troponin levels and normal CT angiography, associated with conditions like takotsubo cardiomyopathy or myocardial infarction with non-obstructive coronary arteries.^65^^,^^66^

The haematocrit from blood draws may not always be available during cardiovascular CT scans. Some studies suggested using attenuation measurements contrast-enhanced dual-energy CT data to calculate a *synthetic* haematocrit.^67^^,^^68^ Calibration for each CT scanner is needed due to iron’s spectral dependency, with scan parameters and monoenergetic reconstruction levels also influencing synthetic haematocrit calculation. Early results on such synthetic ECV calculations show slight under- or overestimation of up to 8% compared to ECV_CT_ using blood haematocrit.^67^ Recently, however, Mergen et al^50^ reported promising results with synthetic haematocrit, showing a mean ECV difference of −0.2% compared to blood haematocrit in patients undergoing dual-source PCD-CT.

Single-energy CT requires both a non-enhanced ECG-gated and a late enhancement scan for ECV_CT_ calculation, assessing contrast media distribution by differences in Hounsfield units (ΔHU) between these scans.^69–71^ However, manual region of interest (ROI) tracing can introduce misregistration errors.^71^^,^^72^ Dual-energy CT scanners improve reliability by enabling spectral separation of late enhancement scans, facilitating material decomposition and iodine quantification, eliminating the need for a non-enhanced scan and simplifying ECV_CT_ calculation.^73^

**Figure 4.**
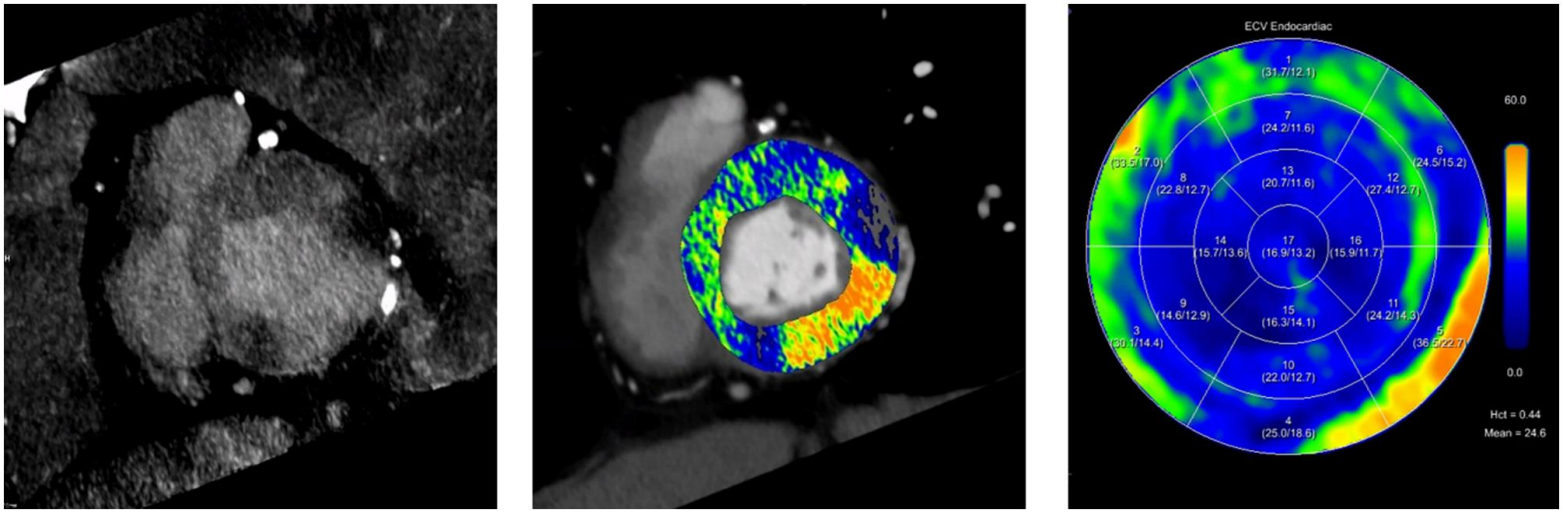
A 83-year-old male patient with severe aortic stenosis undergoing dual-source photon-counting detector CT including a late enhancement scan acquired 5 min after contrast media administration. Iodine map of the late enhancement CT scan and extracellular volume (ECV) polar maps (right) demonstrate pathological late enhancement in the basal inferolateral left ventricular wall. Elevated ECV values are shown in the corresponding territory (max ECV = 36.5 ± 22.7) in overlay (middle) and polar maps (right).

A single spectrally acquired late enhancement scan can simultaneously calculate ECV_CT_ and quantify calcifications using virtual non-enhanced images (VNI). This approach may shift protocols from non-enhanced to standard late-phase acquisitions, offering improved diagnostic and prognostic value at comparable radiation doses.^40^ This is promising for transcatheter aortic valve replacement patients, where studies have shown that ECV_CT_ predicts long-term prognosis in cardiac amyloidosis cases.^74^^,^^75^ PCD-CT provides spectral ECV_CT_ at enhanced spatial and temporal resolution and reduced radiation dose and has therefore the potential for a more wide-spread use of this technique.^76^^,^^77^ Iodine images with ECV maps for late enhancement CT scans are presented in Figure 4.

## Future outlook—multienergy plaque characterization and new contrast media

The quantification and analysis of coronary plaques is of growing importance for risk prediction and clinical patient management.^78^ High-resolution PCD-CT has already demonstrated advantages in plaque quantification and identification of high-risk plaque features.^47^ The addition of spectral information can further refine plaque characterization^79^ by more reliable identification of lipid-rich components. A coveted goal is the visualization of the accumulation of specific contrast agents, such as gold nanoparticles, by refined spectral imaging beyond dual-energy CT (somewhat misleadingly referred to as “k-edge imaging”). K-edge imaging has also been used to selectively display cardiac valve leaflets.^80–82^ Ongoing studies with PCD-CT are focused on further expanding its clinical applications in multienergy imaging, such as advanced plaque characterization and improved coronary assessment. The outcomes of these studies are highly anticipated and expected to further solidify the role of PCD-CT in cardiovascular imaging.^83^

Currently, new CT contrast agents, such as tungsten, are being researched.^84–86^ Unlike iodine, they retain high X-ray attenuation even at high X-ray energies. They are therefore ideal for coronary CT angiography because they can be used to calculate VMIs at high keV to reduce Ca-blooming without compromising the vessel contrast.^84^ In addition, tungsten as a contrast agent facilitates the spectral removal of calcified plaques from the coronary arteries in VNCa images. Table 2 gives an overview on a selection of new contrast agents which are currently under research for potential application in CT.

**Table 2.** Selection of novel contrast agents being the subject of current research for potential application in CT.

Contrast agent	Chemical composition	K-edge (keV)	Possible benefits
Gold	Au (Gold nanoparticles, Heparin coated)	80.7	High contrast in CT, potential for cardiovascular and tumor-specific imaging, prolonged blood circulation time^87^^,^^88^
Tungsten	WO_3_ (Tungsten Oxide)	69.5	Enhanced vessel imaging, reduced blooming artefacts, improved lumen visualization in cardiovascular imaging, interesting dual-energy and monoenergetic properties^84–86^
Platinum	Pt (Albumin-mediated)	78.4	Enhanced angiography, extended contrast duration for cardiovascular imaging, and photothermal therapy^88^^,^^89^
Cerium	CeO_2_ (Cerium Oxide with dextran)	40.4	Antioxidant properties, suitable for imaging in oxidative stress conditions related to cardiovascular disease^88^^,^^90^
Hafnium	Hf (Hafnium-based, BAY-576)	65.4	Improved small vessel imaging and lumen visualization in coronary CT, reduced calcium blooming^85^^,^^91^
Holmium	Ho-DTPA (Holmium)	54.0	High X-ray attenuation, evaluated for coronary imaging but inferior to iodine in some applications^85^^,^^91^
Gadolinium	Gd2O3 (Gadolinium Oxide)	50.2	Dual-modal imaging for CT/MRI, useful for high-contrast anatomical imaging, especially in cardiac and liver imaging^88^^,^^92^

## Conclusion

The recent advent of photon-counting detector CT has triggered further interest in the development and refinement of multienergy cardiovascular CT imaging applications. A short description of the pros and cons of each of the above discussed clinical applications of multienergy cardiovascular CT imaging is provided in Table 3. The combination of high temporal resolution with spectral post-processing represents not only a considerable step forward for current clinical cardiovascular CT but will also spark developments in new contrast media that hopefully will be introduced in the near future, for improving diagnosis and prognostication of our patients.

**Table 3.** Options, advantages and caveats of multienergy cardiovascular CT imaging.

Multienergy option	Advantages	Caveats
Virtual monoenergetic images at lower keV	Enhanced iodine contrast, improved iodine CNR, improved radiation dose efficiency and potential to reduce contrast agent dose at maintained diagnostic accuracy^9^^,^^13–17^	Increased noise at low keV, partially mitigated through advanced processing (Mono+)^17^ Increased blooming from calcified plaques^33^^,^^39^
Virtual monoenergetic images at higher keV	Reduced blooming artefacts from calcified plaques^12^^,^^26^^,^^33^^,^^35–39^^,^^44^ Reduced metal artefacts^33^^,^^35^ Reduced artefacts from dense contrast medium^11^	Reduced iodine contrast Lack of consensus on optimal VMI energy level for coronary plaque visualization and stenosis quantification^36–39^
Virtual non-iodine/virtual non-contrast	Improved radiation dose efficiency and simplified clinical workflow by omitting additional non-contrast Ca-scoring scans^40^^,^^42–44^	Deviation of calcium scores from those of true non-contrast scans, less pronounced with virtual non-iodine images^40^^,^^42–44^
Virtual non-calcium	Aided coronary stenosis quantification by removal of calcified plaques^45^^,^^50^	Lack of robustness with erroneous plaque subtraction in some cases^41^
Late enhancement/extracellular volume quantification	Quantitative myocardial characterization as an alternative to MRI. Increased clinical robustness with dual-source PCD-CT^40^^,^^50^^,^^51^^,^^61–75^	Reduced soft tissue contrast compared to MRI, additional radiation dose^76^^,^^77^
Synthetic haematocrit calculation from blood pool	Obviates the need for drawing blood samples^50^^,^^67^^,^^68^	Synthetic haematocrit calculations can be slightly inaccurate^50^^,^^67^

## Data Availability

No data is available for this manuscript.
